# The prevalence of depression in women with pregnancy‐related pelvic girdle pain: A systematic review and meta‐analysis

**DOI:** 10.1002/hsr2.2308

**Published:** 2024-08-13

**Authors:** Bradley Halliday, Sarah Chatfield, Joanne Hosking, Jennifer Freeman

**Affiliations:** ^1^ Faculty of Health University of Plymouth Plymouth UK; ^2^ Peninsula Clinical Trials Unit Plymouth UK

**Keywords:** depression, pelvic girdle pain, postpartum, pregnancy, systematic review

## Abstract

**Background and Aims:**

Pregnancy‐related pelvic girdle pain (PPGP) is estimated to affect between 20% and 70% of pregnant women with 10% experiencing it for more than 3 months postpartum. Women may also experience depression during this period. Understanding the prevalence of depression in women with PPGP is important to inform clinical management. This systematic review aimed to examine the prevalence of depression in women with PPGP in the antepartum and postpartum periods.

**Methods:**

A systematic review and meta‐analysis. Seven databases were searched from inception until May 24, 2023, combining keywords relating to pelvic girdle pain (PGP), depression, and pregnancy. Two investigators independently screened study titles and abstracts against the eligibility criteria, extracting data characteristics of all included studies. Included articles were assessed for risk of bias. Summary estimates of the prevalence of depression were calculated with a random effects meta‐analysis (stratified by antepartum and postpartum periods).

**Results:**

Eleven studies (3172 participants) were included with nine suitable for meta‐analysis. The overall summary estimate of prevalence of depression among women with PPGP was 24% (95% confidence interval [CI] = 15%–37%), with significant heterogeneity between studies (*I* ^2^ = 97%, *p* < 0.01). Among individual studies, the estimates ranged from 18% to 48% in the antepartum PGP population and from 5% to 39% in the postpartum PGP population. The summary estimate in the antepartum group was 37% (95% CI = 19%–59%; prediction interval 8%–81%) and 15% (95% CI = 7%–30%; prediction interval 3%–56%) in the postpartum group, although time (antepartum vs. postpartum) did not have a statistically significant moderating effect (*p* = 0.06). Two thirds of the studies were undertaken with Scandinavian populations, limiting the generalizability of these findings.

**Conclusion:**

Summary estimates for the prevalence of depression in women with PPGP are similar to previous studies investigating depression in the general peri‐natal population.

## 1 INTRODUCTION

Pregnancy‐related pelvic girdle pain (PPGP) is experienced by 20%–70% of women during the antenatal period^1^, ^2^ and up to 6 weeks after delivery,^3^, ^4^ depending on the methods of assessment. It is characterized by pain experienced between the posterior iliac crest and the gluteal fold, which can be experienced in conjunction with/or separately in the symphysis.^5^ By 3 months postpartum many will have recovered, however, 10% continue to experience PPGP^6^ seeking interventions from physiotherapists to manage this.^7^ Persistent PPGP can be difficult to treat, with evidence of the condition's presence up to 12 years postpartum,^8^ despite the dogma that this is a self‐limiting condition, resolving soon after delivery.^9^

Musculoskeletal pain and depression are often encountered together and may co‐exacerbate physical and psychological symptoms.^10^ Depression is characterized by the absence of positive affect, low mood, and a range of associated cognitive, behavioral, physical, and emotional symptoms.^11^ It is a leading cause of disability and key contributor to the global burden of disease^12^ with a lifetime prevalence ranging between 20% and 25% for women.^13^

Women are at the greatest risk of depression during their childbearing years, being three times more likely than men to experience depression during this time.^14^ Despite the perinatal period often being a period of positive expectation and happiness, it is a time of vulnerability^15^ with a range of new challenges such as a reduction in sleep,^16^ being associated with the risk of mental health problems,^17^ alongside physical and psychological changes.^18^ The multiplicity of these factors makes it challenging to specifically determine whether PPGP may further influence rates of depression compared to the general peri‐natal population. This is important since depression is thought to be a potential contributory factor to the persistence of chronic PPGP.^19^

Varying prevalence rates are reported in published studies of women with PPGP. For example, the point prevalence for depression in the antepartum period varies between 15% and 30%,^20^, ^21^ with postpartum estimates in a similar, albeit narrower range between 17% and 18%.^20^, ^22^, ^23^, ^24^, ^25^

Understanding the prevalence of depression in PPGP is important to physiotherapists and other health and care professionals for several reasons^26^: to frame the service needs of this population; as a factor to consider when implementing healthcare interventions such as physiotherapy; ensuring adequate resource allocation to this population; and in the design of clinical trials (support/safeguards and intervention design). Moreover, depression can have a significant impact, not only on the mother but also on the development and health of infants, resulting in poorer cognitive and emotional development.^27^, ^28^ In addition, there is a need to ensure that women are equally served by research^29^ and that problems that primarily affect women are further understood.^30^, ^31^

Therefore, the aim of this systematic review and meta‐analysis is to synthesize and critically appraise the literature and provide a summary estimate of the prevalence of depression, for women with PPGP in both the antenatal and postnatal periods. To our knowledge, there is no previous review addressing this question.

## 2 MATERIALS AND METHODS

### 2.1 Search strategy and selection criteria

This systematic review followed the approach recommended by the Preferred Reporting Items for Systematic Reviews and Meta‐analyses guidelines.^32^

The MEDLINE, PubMed, Embase, CINAHL, AMED, PsycINFO, and Web of Science databases were initially searched from source inception to June 29, 2022. Secondary electronic searches were also conducted in ProQuest Dissertations and Theses Global, Pedro, the Cochrane Central Register of Controlled Trials (CENTRAL), United States National Library of Medicine Registry of Clinical Trials, World Health Organization International Clinical Trials Registry Platform portal, and Epistemonikos. An updated search of all the databases above was completed on May 24, 2023 to allow for any additional publications since the initial search. Grey literature searching was also completed in a range of websites, archives, Ebooks, and conference abstracts. Electronic searches were supplemented by hand searches of reference lists, as described in the protocol.^33^

There was no restriction on language and a translation tool (Google Translate) was used when sources were not published in English. Except for study protocols, any study design was considered for inclusion.^34^ Systematic and scoping reviews were included to ensure that all relevant studies were considered. The search strategy used the CoCoPop approach (Condition, context, and population)^35^ and combined terms related to pelvic girdle pain (PGP), depression, and pregnancy. Full search details are provided in Supporting Information S1: File 1. Articles were selected for inclusion where the prevalence of PPGP and depression were presented or could be calculated from available data. Population and condition were adult women ≥18, experiencing PPGP at any time during pregnancy, and/or at any time postpartum. PPGP was defined as PGP that started during pregnancy or up to 6 weeks postpartum).^3^, ^4^ PGP could be self‐reported, or clinician rated. Depression was determined by assessment tools to indicate depression (validated patient‐reported or clinician‐rated outcome measures).

Articles were excluded on the following criteria: women under 18, low mood which is not defined as depression, onset of PGP >6 weeks post‐delivery, and not including a group with PPGP.

The titles and abstracts of retrieved articles were initially screened against the inclusion criteria and irrelevant articles were excluded at this point. The full text was sought for the remaining articles to identify those that met the inclusion criteria.

### 2.2 Data extraction

A bespoke data extraction form was used by two authors independently to extract equivalent data from each included article. The following information was extracted: Author, title, year of publication, country of paper, geography of institutions involved, study design, aims and objectives, care setting, sample size at baseline, study dropout, numbers analyzed at study completion, age of participants, data collection procedures, outcome measures, numbers with depression, numbers with PGP. Regarding depression, the definition of depression reported, screening assessment used, and timing of the depression assessment were extracted. Regarding PGP, the definition of PGP, how it was assessed, the participant's diagnosis in terms of PGP, duration of PGP, pain intensity, and severity were extracted.^36^

### 2.3 Risk of bias

All included articles were assessed for risk of bias by two reviewers using the Joanna Briggs Institute prevalence checklist.^37^ This ensured consistency in assessment due to the diversity of study designs reporting prevalence data.^38^ This review has not excluded papers based on methodological quality.

### 2.4 Reliability

Two investigators (either BH, SC or JF) independently screened article titles, abstracts and where relevant full‐text articles for inclusion and undertook data extraction and risk of bias assessments.^32^ Differences in opinion were discussed between the two authors, with the third available to mediate any issues that were unresolved.

### 2.5 Statistical analysis

A meta‐analysis was performed to examine the prevalence of depression among women with PGP. The studies were split according to the time at which depression was assessed, that is, either antepartum or postpartum. The raw proportions from each study were transformed by the logit transformation. A random effects model was then used to combine studies within each subgroup and to produce an overall estimate, assuming a common within‐group heterogeneity across the two subgroups. Two interval estimates were calculated: a confidence interval (i.e., precision of the estimate), and prediction intervals, indicating the range into which estimates from future original studies are likely to lie.^39^ A sensitivity analysis was undertaken whereby two studies of unclear risk of bias in the antepartum group^40^, ^41^ were removed to see whether and how this affected the summary estimate of prevalence. The “meta” and “metafor” packages in R were used for analysis.^42^, ^43^, ^44^

### 2.6 Systematic review registration

The protocol for this systematic review was registered at PROSPERO International Prospective Register of Systematic Reviews 2017 (record: CRD42022342575) Register 29 June 2022.

## 3 RESULTS

### 3.1 Included studies

**Figure 1:**
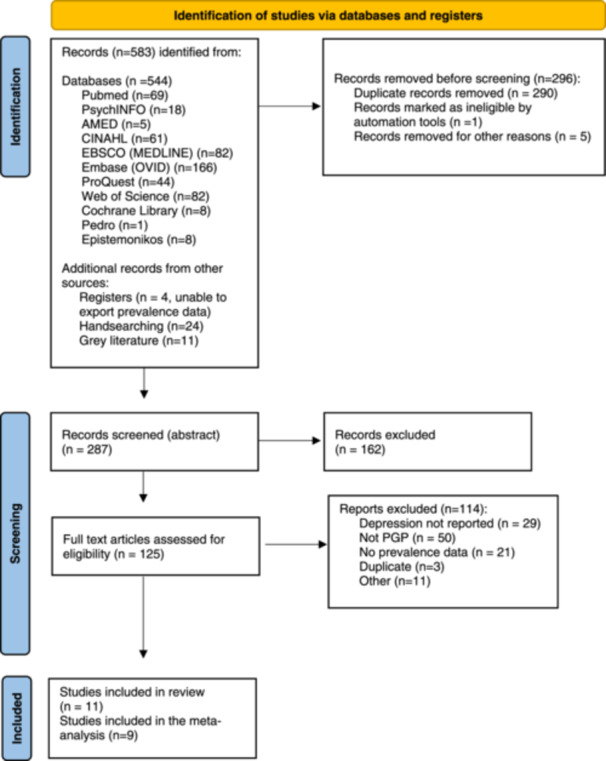
Preferred Reporting Items for Systematic Reviews flow diagram of study inclusion. PGP, pelvic girdle pain.

The Preferred Reporting Items for Systematic Reviews and Meta‐Analyses (PRISMA) flowchart (Figure 1) provides details regarding study inclusion.^45^ 583 papers were identified from all searches completed. 125 articles were included for full‐text screening, of which 114 were excluded if depression data were not reported, PGP not reported, no prevalence data were available, or the article was a duplicate. Eleven studies met the inclusion criteria and were therefore included for data extraction. Two of these studies were not included in the meta‐analysis. The first^46^ was a qualitative study of postpartum women (*n* = 9) 3 months after birth. It was not clear whether the reported depression in the participants (*n* = 5) was related to the antepartum or postpartum period. The second^2^ was a scoping review and included two studies of relevance to the prevalence of depression in PPGP, however, both studies were already identified individually in the search strategy and hence were not included to avoid double counting.

These involved 3172 women and had a range of designs: three cohorts, two cross‐sectional, two case–control, and two longitudinal questionnaires (Tables 1 and 2).

**Table 1:** Summary of included antepartum studies.

Author	Country	Study design	Women with PGP (*n* = 2541)	Women with PGP and depression (*n* = 692)	Depression screening tool	Depression screening (time point)	PGP assessment approach
Dørheim et al.^47^	Norway	Longitudinal Questionnaire study	1707	312	EPDS ≥10	Week 32	Self‐report—Screened via two questions: experienced long lasting or frequently recurring pain during the last 2 weeks (coded yes/no); pain in the pelvic girdle (coded yes/no), and if so, location of the pain (frontal part of the pelvis, one side of the rear part, or both sides of the rear part of the pelvis)
Kovacs et al.^1^	Spain	Cross‐sectional	742	339 (slightly depressed *n* = 266; moderately depressed *n* = 61; seriously depressed *n* = 12)	BDI slightly depressed 10–18	28 weeks of pregnancy	Self‐report via a single question: In the past 4 weeks, have you had pain in your low back area, down the leg, or in the pelvic area (as shown on a drawing representing a human body).
					Moderately depressed 19–29		
					Seriously depressed ≥30		
Malmqvist et al.^40^	Norway	Longitudinal cohort	63	27	Dichotomous Feeling depressed (Yes/No)	Week 18	Objective assessment. Tests included in the following sequence: Active straight leg raise, Gaenslens test, Long Dorsal sacroiliac ligament test, Modified Trendelenburg's test, FABER test, P4, Symphysis palpation test
Meucci et al.^41^	Brazil	Cross‐sectional	29	14	Not reported	Within period of pregnancy	Self‐report: The presence of PGP was classified according to the location of pain indicated on a figure.

Abbreviations: BDI, Beck Depression Inventory; EPDS, Edinburgh Postnatal Depression Scale; FABER, Flexion, Abduction, External Rotation; PGP, pelvic girdle pain.

**Table 2:** Summary of included postpartum studies.

Author	Country	Study design	Women with PGP (*n* = 631)	Women with PGP and depression (*n* = 76)	Depression screening tool	Depression screening (time point)	PGP assessment approach
Gutke et al.^48^	Sweden	Cohort	44	17	EPDS ≥10	3 months postpartum	Objective assessment: 2 or more positive pelvic pain provocation tests from; Distraction test, P4, Gaenslens test, Compression test, sacral thrust. Absence of centralization or peripheralization phenomena during repeated movement assessment. No lumbar effect from repeated movement according to the MDT classification. Pain onset during pregnancy or within 3 weeks after delivery.
Elden et al.^49^	Sweden	Longitudinal follow‐up	37	2	HADS—cutoff of 8/21 for depression	Postpartum period, time points not stated	Objective assessment:
							The following criteria applied for a diagnosis of PGP: Pain experienced between the posterior iliac crest and the gluteal fold, particularly in the vicinity of the SIJ in conjunction with/or separately in the symphysis. Reports of duration and weight‐bearing‐related pain in the pelvic girdle. Diminished endurance in standing, walking, and sitting. 2 or more positive clinical diagnostic tests from: posterior pelvic pain provocation test (P4), distraction test, compression test, sacral thrust, and MAT test. No nerve root syndrome. No reproducible pain and/or changed symptoms in the lumbar spine by repeated end range movement.
Ertmann et al.^50^	Denmark	Prospective cohort	436	38	MDI score >20	8 weeks postpartum	Self‐report: the presence of PGP was classified according to the location of pain indicated on a figure (answers were categorized as (yes/no).
Long et al.^51^	China	Retrospective case–control	68	7	EPDS ≥13	6 months postpartum	Objective assessment: A diagnosis of PGP was made if women had: A pain drawing with markings in the gluteal area, time‐ and weight‐bearing related to pain deep in the gluteal area, pain‐free intervals, free range of motion in the spine, and a positive result on the posterior pelvic pain provocation test.
Rexelius et al.^52^	Sweden	Case–control	46	12	MADRS‐S ≥13	Postpartum period (3–12 months)	Objective assessment: A diagnosis of PGP was made if women reported sacral pain with an onset in pregnancy and at least one positive pain provocation test of either, P4 test, Menell's test or FABER test and experience pain with pressure on the ischial spine ipsilaterally to reported pain on at least one side.

Abbreviations: EPDS, Edinburgh Postnatal Depression Scale; FABER, Flexion, Abduction, External Rotation; HADS, Hospital Anxiety and Depression Scale; MADRS‐S, Self‐rated Montgomery‐Asberg Depression Rating Scale; MDI, Major Depression Inventory; MDT, Mechanical Diagnosis and Therapy; P4, posterior pelvic pain provocation test; PGP, pelvic girdle pain; SIJ, sacroiliac joint.

### 3.2 Risk of bias

**Figure 2:**
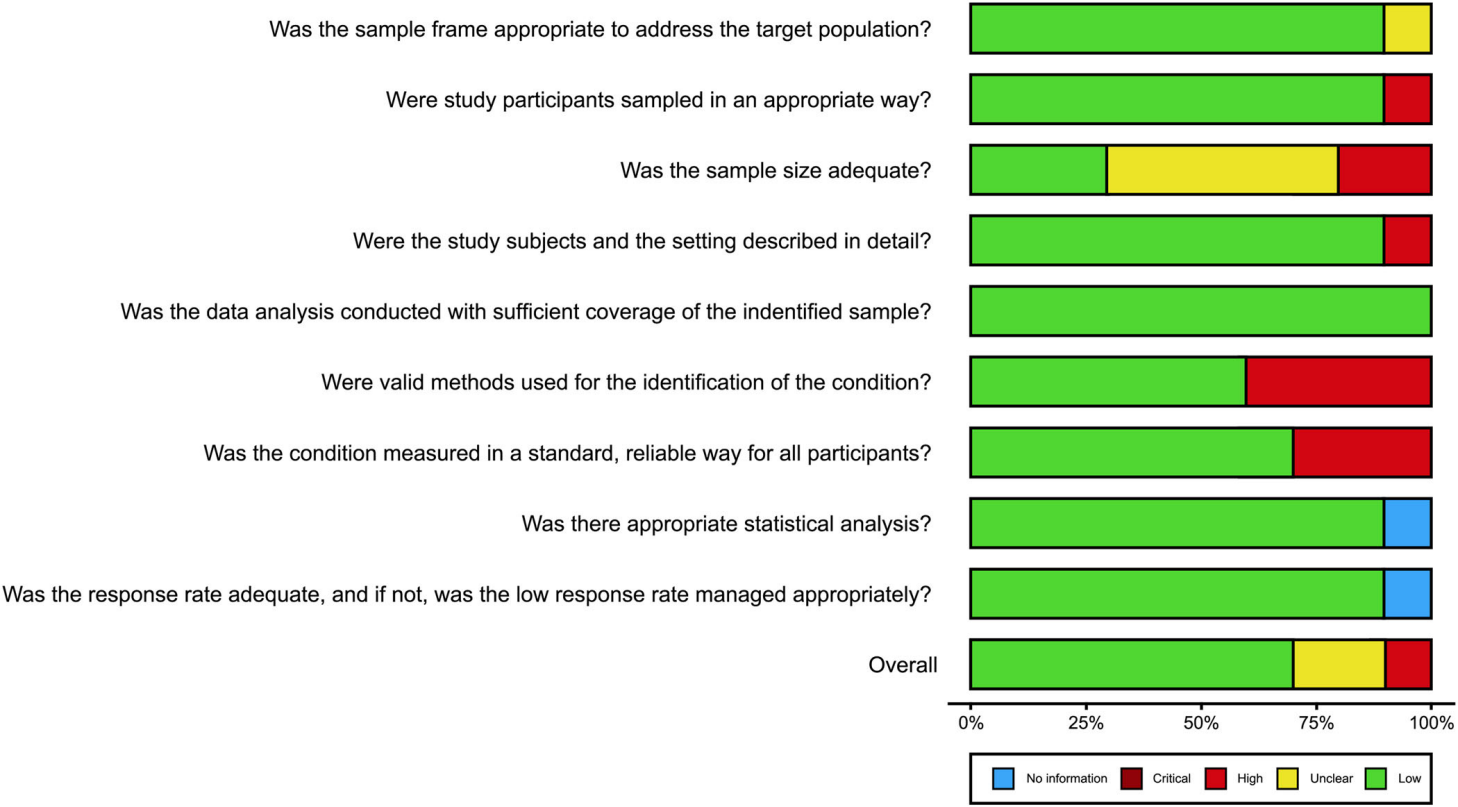
Joanna Briggs Institute risk of bias assessment—summary plot. Overall quality: green—good, yellow—medium, and red—poor.

**Figure 3:**
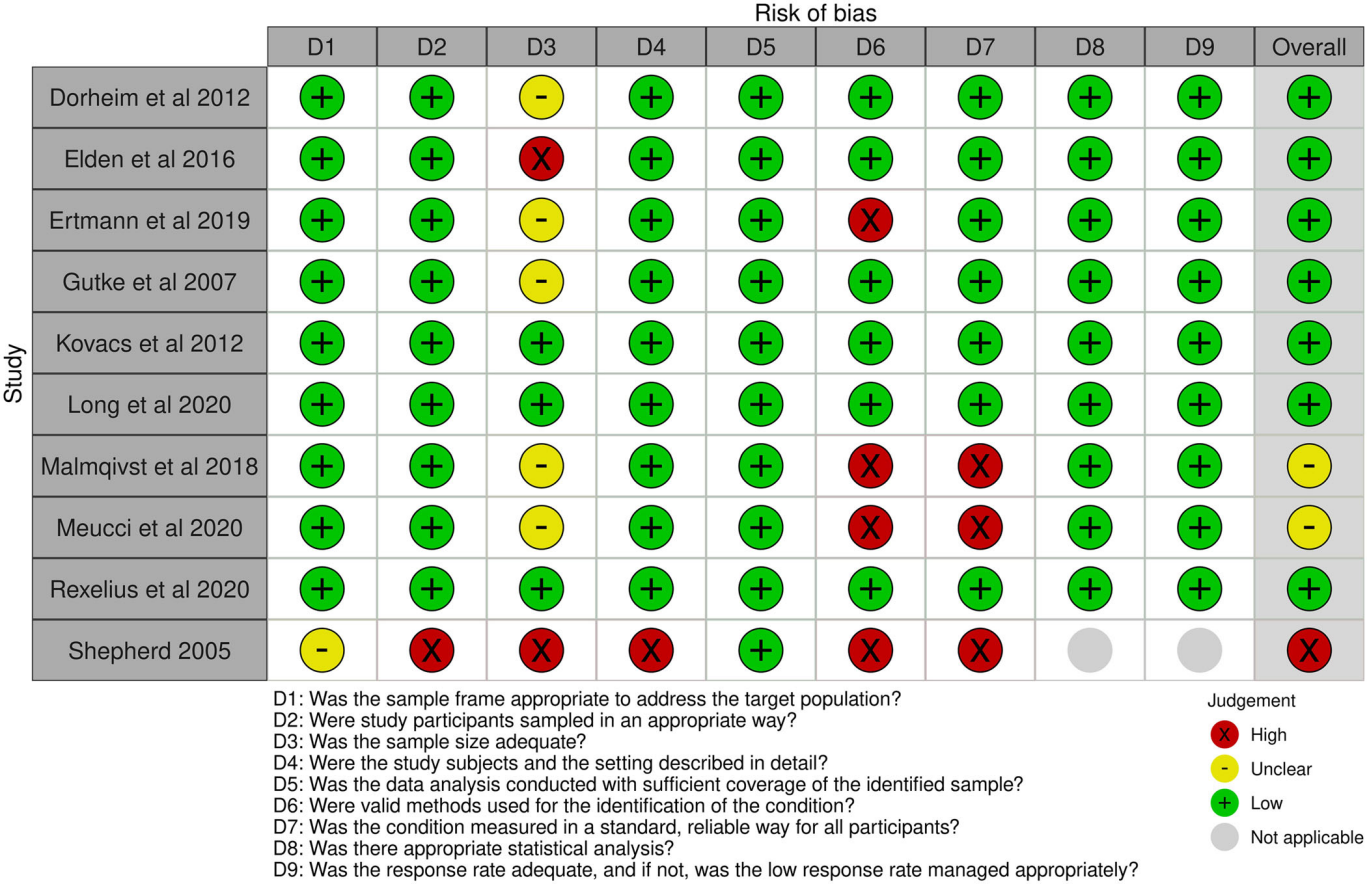
Joanna Briggs Institute risk of bias assessment—individual papers.

The risk of bias assessment outcomes are detailed as a summary plot (Figure 2), demonstrating that overall, the included studies were of low risk of bias.^37^ Domains with the highest risk of bias were related to valid methods for the identification or measurement of the condition with two studies failing to use a standardized screening assessment for depression, and one failing to measure depression in a standard and reliable way. In addition, seven studies did not report on sample size estimates and therefore were rated as unclear. Risk of bias assessment results for individual papers are detailed in Figure 3.

### 3.3 Study characteristics

Key characteristics of those studies included in the meta‐analyses are detailed in Tables 1 and 2.

### 3.4 Prevalence of depression in PPGP

**Figure 4:**
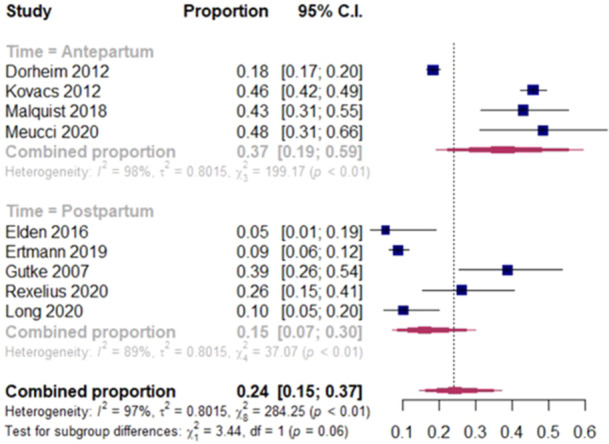
Meta analysis of the prevalence of depression in pregnancy‐related pelvic girdle pain. C.I. confidence interval.

The results for each study estimate for the two subgroup summary proportions and the overall summary proportion are displayed in the forest plot in Figure 4. Among individual studies, the estimates ranged from 0.18 to 0.48 in the antepartum PGP population and from 0.05 to 0.39 in the postpartum PGP population. The summary estimate of the proportion in the antepartum period was 0.37 (95% confidence interval (CI) = 0.19–0.59; prediction interval = 0.08–0.81) and 0.15 (95% CI = 0.07–0.30; prediction interval = 0.03–0.56) in the postpartum period although time (antepartum vs. postpartum) did not have a statistically significant moderating effect (*p* = 0.06) and the overall summary estimate of the proportion was 0.24 (95% CI = 0.15–0.37; prediction interval = 0.03–0.75). The overall *I* ^2^ statistic was 97% indicating a high degree of heterogeneity. Removing the two studies of unclear risk of bias in the antepartum group^40^, ^41^ lowered the summary estimate of prevalence to 0.30 (95% CI = 0.11 to 0.61), see Supporting Information S1: File 2.

### 3.5 Geography

Overall, six studies^40^, ^47^, ^48^, ^49^, ^50^, ^52^ originated from Scandinavia with the remaining originating from Brazil,^41^ China,^51^ Spain^1^ and the United Kingdom (UK).^46^ Of the five studies investigating postpartum depression, four originated from Scandinavia,^48^, ^49^, ^50^, ^52^ and one study from China.^51^ In terms of geography, there was a greater diversity in the antepartum group with two studies from Scandinavia and the remaining two studies from Spain and Brazil.

### 3.6 Screening tools for depression

A range of methods to screen for depression were used. In the antepartum population, no study used the same outcome measure. Two studies used a standardized and validated measure: the Edinburgh Postnatal Depression Scale (EPDS),^47^ which does not categorize the severity of depression, and the Beck Depression Inventory (BDI)^1^ with this latter study differentiating the severity of the depression from slightly depressed to seriously depressed. Data included in the meta‐analysis were from all severities of depression (Table 1). Two studies did not use a validated measure^40^, ^41^ and no study assessed depression at the same time point.

In contrast, all included studies in the postpartum population used a standardized and validated screening tool. Two studies used the same measure (EPDS), however with different thresholds for depression.^48^, ^51^ One study^52^ used the Self‐rated Montgomery‐Asberg Depression Rating Scale (MARDS), being the most extensively used instrument in depression research after a categorical diagnosis has been made.^53^ The threshold used in this study was ≥13 which encompasses varying degrees of depression.^54^ One study^49^ used the Hospital Anxiety and Depression Scale (HADS), which does not categorize the severity of depression. One study used the Major Depression Inventory (MDI),^50^ which can categorize severity; a threshold >20/50 was used, including a range of depression from mild to severe.^55^ No study assessed depression at the same time point, with time points ranging from 3 to 12 months postpartum.

### 3.7 Assessment of pelvic girdle pain

The assessment of PPGP varied throughout the studies. In the antepartum population, only one study utilized an objective assessment to diagnose PGP^40^ using a range of tests (Table 1). The threshold of positive tests for a diagnosis of PGP was not stated. All other studies utilized self‐report measures which comprised a range of questions and pain drawings.

In contrast, in the postpartum population, four studies utilized an in‐person objective assessment of PGP through a range of recognized pain provocation tests.^48^, ^49^, ^51^, ^52^ Two studies^48^, ^49^ used the same testing battery in the order of distraction test, Posterior Pelvic Pain Provocation Test (P4), Gaenslens, compression, sacral thrust with a threshold of ≥2 positive pain provocation test for the diagnosis of PPGP. Elden et al.,^49^ also included the MAT test to screen for anterior pelvic girdle pain. One study^52^ used a lower threshold of at least one positive pain provocation test from: Posterior Pelvic Pain Provocation Test (P4), Flexion, Abduction, External Rotation (FABER), and pain provocation from gentle pressure on the ischial spine on at least one side. One study used a single physical test to assess the presence of PGP, the P4 test.^51^

## 4 DISCUSSION

This systematic review and meta‐analysis demonstrated that the prevalence of depression in antepartum and postpartum populations with PPGP is similar to the prevalence of depression in the general peri‐natal populations without PPGP; 15%–30% in the general antepartum population^20^, ^21^ compared to our summary estimate of 37% in the PPGP population, and 17%–18% in the general postpartum population^20^, ^22^, ^23^, ^24^, ^25^ compared to our summary estimate of 15% in the PPGP population. This indicates that PPGP may not be associated with an increased risk of depression, antenatally or postnatally, although this must be taken within the context of the limitations of the review.

The limited volume, quality, and variation between studies in this area suggest that further research is needed to better understand the relationship between PPGP and depression. Of note, a recent study found the symptoms of depression in early pregnancy were associated with the development and intensity of PPGP later in pregnancy.^56^ Other factors may also be associated with the onset of depression including a range of obstetric and psychosocial factors^57^ which may influence central mechanisms of persistent pain.^58^ Studies included in this review did not indicate the presence of pre‐existing depression and were cross‐sectional in nature, therefore did not allow exploration of the temporal relationship between PPGP and depression. These considerations are important for informing physiotherapy practice.

Our protocol did not state methodological quality as an eligibility criterion, with all published studies included to provide a comprehensive review. Methodological quality in the studies varied, and two studies^40^, ^41^ were graded as a medium risk of bias (Figure 3), overall indicating lower methodological quality. There is therefore a risk of bias in their results, that is, they may overestimate or underestimate the true incidence of depression. We therefore undertook a sensitivity analysis to determine their influence on the summary prevalence estimates; demonstrating their impact on the estimate. The overall certainty of evidence is low for studies included within the meta‐analysis and conclusions drawn should therefore be tentative.

The majority of studies included in this review were based in Scandinavian countries, particularly those focused on postpartum PPGP. One other study from Europe^1^ (Spain) was included in the meta‐analysis. Therefore a lack of knowledge exists on the prevalence of depression in women with PPGP from other European countries. Only one study was based within the UK,^46^ which was unable to provide robust estimates of prevalence, having a high risk of bias, a small sample size (*n* = 9), and a lack of clarity on the period of self‐reported depression. Culture and ethnicity have a multi‐faceted influence on depression^59^, ^60^, ^61^, ^62^ and hence the geography of the study may be an important factor affecting the prevalence estimates provided, with a dominance of studies from Scandinavia in this review. A greater level of support offered to Scandinavian women, relative to some countries^63^ and a greater awareness of the condition^64^ leading to earlier management may affect the prevalence estimates provided and reduce the generalizability of the results.

PPGP has been recognized to have an onset, primarily in the antepartum, but also postpartum period. A range of definitions are used for the postpartum period.^65^, ^66^ This review used the World Health Organization,^4^ definition which considers this period to last 6 weeks. The results may have been different if an alternative timeframe was used.

Accurate estimates of prevalence are dependent on a consistent and recognized method of assessment, in this case for both PGP and depression. Current European Guidelines for the assessment of PGP^5^ recommend the use of a physical examination using a battery of pain provocation tests. The postpartum studies consistently used a combination of recommended objective tests to confirm the presence of PGP. Therefore, there is a greater level of certainty that this population had PPGP, and depression having also used a validated measure of depression. The reverse is true for the antepartum population, where only one study included a physical examination to determine the presence of PGP and only used a dichotomous Yes/No for defining the presence of depression. A more accurate prevalence estimate would require studies examining this population to collect data on depression using standardized and validated measures, as has been achieved in a recent feasibility trial in the UK.^67^ However, single measures alone, such as the EPDS, may not be suitable for the detection of major depressive disorders, particularly during pregnancy.^68^

Of the studies included in this review, only three reported on pain severity, typically using a Visual Analog Scale. Where pain severity is reported, there is no indication of the severity of depression, except in one study^1^; however, the relationship between these factors was not examined in this study. It would be beneficial for future studies to report the relationship between the severity of pain and depression to determine the magnitude and significance of such a relationship.^69^

Only 11 studies met the inclusion criteria for this review, suggesting a lack of research in this area. There are several possible reasons for this. PGP is often not seen as a discrete condition from LBP^70^, ^71^ and therefore does not achieve particular attention in research. Furthermore, women are an underrepresented group in research^29^ and may not be exposed to the same research opportunities as their male counterparts. Finally, women may not seek support, particularly in the postnatal period, due to many factors.^70^, ^71^, ^72^, ^73^ For example, in previous studies, women have reported feelings of having to “put up with” persistent pain, adapt their daily activities accordingly to meet the demands of their new baby and feeling “brushed off” by care providers^70^, ^71^ with evidence of dissatisfaction in the UK with the 6‐ to 8‐week postpartum check.^74^, ^75^ With these experiences considered, many women may be left attempting to self‐manage persistent pain following childbirth, due to fears of not being listened to or helped. In addition, women may prioritize the health of their baby over their own health, finding it difficult to find time to help themselves.^76^

To our knowledge, this is the first systematic review on the prevalence of depression in women with PPGP and therefore offers an initial, albeit somewhat limited, insight into the prevalence. This review has utilized a consistent and recommended tool for the assessment of risk of bias for prevalence studies and included prediction intervals to demonstrate heterogeneity.^77^ There was significant heterogeneity between studies with a range of depression measures and methods for the assessment of PPGP. This highlights a need for future research to utilize recommended methods to assess the presence of PPGP and depression, particularly in the antepartum period. The majority of studies were from Scandinavia, highlighting a need to better understand the prevalence of depression in women with PPGP in other countries. Therefore, caution is suggested in the interpretation of the results. There is a notable absence of studies from the UK, America, Australia, Asian, and African continents. This may be due to several factors, which include a lack of awareness of PGP in expectant and new mothers^78^ and healthcare practitioners.^70^, ^71^ A greater recognition of PGP as a discrete musculoskeletal condition is needed to reflect more accurately the prevalence of PGP and depression, so that this can be taken into consideration with service provision.

## 5 CONCLUSION

This systematic review has provided summary estimates of depression specifically in women with PPGP. The estimates range from 18% to 48% in the antepartum PGP population and 5% to 39% in the postpartum PGP population. These estimates are similar to the prevalence of depression in the general peri‐natal populations. These estimates must be interpreted in relation to the limitations discussed in the review. There is a need for future research to include standardized screening measures for depression, and where relevant PGP, in antepartum and postpartum populations to gain a more reliable estimate of prevalence.

## AUTHOR CONTRIBUTIONS

**Bradley Halliday**: Conceptualization; data curation; formal analysis; investigation; methodology; project administration; resources; visualization; writing—original draft; writing—review and editing. **Sarah Chatfield**: Conceptualization; data curation; formal analysis; Investigation; methodology; project administration; resources; visualization; writing—original draft; writing—review and editing. **Joanne Hosking**: Formal analysis; funding acquisition; methodology; resources; visualization; writing—review and editing. **Jennifer Freeman**: Conceptualization; funding acquisition; methodology; project administration; resources; validation; visualization; writing—review and editing.

## CONFLICT OF INTEREST STATEMENT

The authors declare no conflict of interest.

## TRANSPARENCY STATEMENT

The lead author Bradley Halliday affirms that this manuscript is an honest, accurate, and transparent account of the study being reported; that no important aspects of the study have been omitted; and that any discrepancies from the study as planned (and, if relevant, registered) have been explained.

## Supporting information

Supporting information.

Supporting information.

Supporting information.

## Data Availability

This study did not involve the use of a human research database, so data sharing is not applicable. Meta‐analysis data were extracted from articles found in the literature.
