# Supplementary material for: The prevalence of depression in women with pregnancy‐related pelvic girdle pain: A systematic review and meta‐analysis
Source: Health Sci Rep. 2024 Aug 13;7(8):e2308. doi: 10.1002/hsr2.2308 (PMC11322010; doi:10.1002/hsr2.2308)
Supplement: Supplementary file 1 — Supporting information. [file HSR2-7-e2308-s002.docx]

SUPPLEMENTARY FILES

Supplementary file 1 – CoCoPop search terms

| **Pelvic girdle pain**    **(Concept 1)** | **AND** | **Depression**    **(Concept 2)** | **AND** | **Pregnancy related**    **(Concept 3)** |
| --- | --- | --- | --- | --- |
| “pelvic girdle pain” OR pgp OR “pelvic girdle dysfunction” OR “pelvic girdle relaxation” OR “low back pain” OR “pelvic instabilit*” OR “pelvic insufficienc*” OR “pelvic relaxation” OR “posterior pelvic pain” OR “peripartum pelvic pain” OR “postpartum pelvic pain” OR “symphysis pain” OR “symphysis dysfunction” OR “pubic symphysis pain” OR “pubic symphysis dysfunction” OR “symphysis pubic pain” OR “symphysis pubic dysfunction” OR “lumbopelvic pain” OR lpp OR “sacroiliac joint pain” OR “sacroiliac joint dysfunction” OR symphysiolysis OR “sacral pain” |  | Depress* |  | Pregnan* OR antenatal OR antepartum |

Supplementary file 2 - Meta analysis of the prevalence of Pregnancy related Pelvic Girdle Pain.


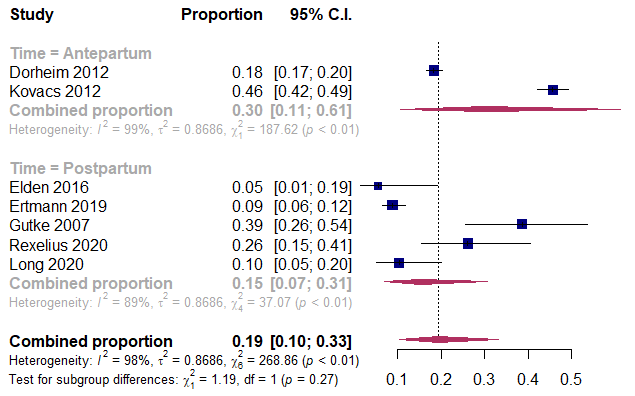


Note: Two studies with a higher risk of bias excluded from the antepartum subgroup analysis.

Legend: C.I. confidence interval
